# Distal Appendicitis

**Published:** 1913-11

**Authors:** 


Distal Appendicitis. Boekel, *La Rif. Med.*, November 12, 1912, describes what he considers a new form of appendicitis, in which the tip becomes adherent to the mesentery and discharges between its folds, or between loops of intestine, forming a dense mass or masses consisting of pockets of pus. In one case he saved the patient by resecting 80 c.m. of ilium and 90 c.m. of cæcum and ascending colon. (Note. We are inclined to believe that this type has been recognized by American surgeons and that the extraordinary length of large intestine mentioned represents a misprint.)

